# Microbial diversity of ticks and a novel typhus group *Rickettsia* species (*Rickettsiales* bacterium Ac37b) in Inner Mongolia, China[Fn FN1]

**DOI:** 10.1051/parasite/2023057

**Published:** 2023-12-12

**Authors:** Si Su, Mei Hong, Meng-Yu Cui, Zheng Gui, Shi-Fa Ma, Lin Wu, Li-Li Xing, Lan Mu, Jing-Feng Yu, Shao-Yin Fu, Rui-Juan Gao, Dong-Dong Qi

**Affiliations:** 1 Graduate School, Inner Mongolia Medical University Hohhot 010059 Inner Mongolia China; 2 School of Basic Medicine, Inner Mongolia Medical University Hohhot 010110 Inner Mongolia China; 3 First Hospital of Jilin University Changchun 130021 China; 4 Hulunbuir Mental Health Center Hulunbuir 022150 Inner Mongolia China; 5 Beijing Guoke Biotechnology Co., Ltd 102200 Beijing China; 6 Department of Infection Control, Second Affiliated Hospital of Inner Mongolia Medical University Hohhot Inner Mongolia Autonomous Region 010000 China; 7 Inner Mongolia Academy of Agricultural & Animal Husbandry Science Hohhot 010031 Inner Mongolia China

**Keywords:** Inner Mongolia, *Dermacentor nuttalli*, *Ixodes persulcatus*, Microbial diversity, *Rickettsiales* bacterium Ac37b

## Abstract

Ticks can carry multiple pathogens, and Inner Mongolia’s animal husbandry provides excellent environmental conditions for ticks. This study characterized the microbiome of ticks from different geographical locations in Inner Mongolia; 905 *Dermacentor nuttalli* and 36 *Ixodes persulcatus* were collected from sheep in three main pasture areas and from bushes within the forested area. Mixed DNA samples were prepared from three specimens from each region and tick species. Microbial diversity was analyzed by 16S rRNA sequencing, and α and β diversity were determined. The predominant bacterial genera were *Rickettsia* (54.60%), including *Rickettsiales* bacterium Ac37b (19.33%) and other *Rickettsia* (35.27%), *Arsenophonus* (11.21%), *Candidatus* Lariskella (10.84%), and *Acinetobacter* (7.17%). *Rickettsia bellii* was identified in *I. persulcatus*, while *Rickettsiales* bacterium Ac37b was found in *D. nuttalli* from Ordos and Chifeng. Potential *Rickettsia* and *Anaplasma* coinfections were observed in the Ordos region. Tick microbial diversity analysis in Inner Mongolia suggests that sheep at the sampling sites were exposed to multiple pathogens.

## Abstract (fr)

Les tiques peuvent être porteuses de plusieurs agents pathogènes et l’élevage en Mongolie intérieure offre d’excellentes conditions environnementales pour les tiques. Cette étude a caractérisé le microbiome des tiques de différentes zones géographiques de Mongolie intérieure; 905 *Dermacentor nuttalli* et 36 *Ixodes persulcatus* ont été collectés sur des moutons dans trois principales zones de pâturage et dans des buissons de la zone forestière. Des échantillons d’ADN mixtes ont été préparés à partir de trois spécimens de chaque région et espèce de tique. La diversité microbienne a été analysée par séquençage de l’ARNr 16S et la diversité α et β a été déterminée. Les genres bactériens prédominants étaient les *Rickettsia* (54,60 %), dont la bactérie *Rickettsiales* Ac37b (19,33 %) et d’autres *Rickettsia* (35,27 %), *Arsenophonus* (11,21 %), *Candidatus* Lariskella (10,84 %) et *Acinetobacter* (7,17 %). *Rickettsia bellii* a été identifiée chez *I. persulcatus*, tandis que la bactérie *Rickettsiales* Ac37b a été trouvée chez *D. nuttalli* d’Ordos et Chifeng. Des co-infections potentielles à *Rickettsia* et *Anaplasma* ont été observées dans la région d’Ordos. L’analyse de la diversité microbienne des tiques en Mongolie intérieure montre que les moutons présents sur les sites d’échantillonnage sont exposés à plusieurs agents pathogènes.

## Introduction

Ticks are arachnids (Arachnida: Acari: Ixodida) that parasitize birds, reptiles, amphibians, and terrestrial mammals, including humans. Ticks are bloodsucking at all developmental stages. When biting their hosts, ticks can be vectors for the transmission of various pathogens and cause irritation, anemia, and local or systemic hypersensitivity [37]. Ticks are second only to mosquitos as human disease vectors throughout the world [8, 19, 68]. They can transmit numerous pathogens, such as viruses, bacteria, and protozoa [32]. *Rickettsia* are vertically transmitted symbionts in invertebrates and pathogens in vertebrates. Tick-borne rickettsioses are caused by obligatory intracellular bacteria from the spotted fever group of the genus *Rickettsia* [43]. In the past few decades, tick-borne *Rickettsia* infections have been reported worldwide, most of which are widely distributed in tropical and subtropical areas, and the scope of infection is expanding. Although some *Rickettsiales* isolates are not associated with pathogenicity in vertebrates, thirteen tick-borne *Rickettsiales* pathogens have been reported to cause human disease in China, including seven spotted fever group *Rickettsia* (SFGR), two species of *Ehrlichia*, and four other species in the family Anaplasmataceae [12, 14]. Ticks are found all across China; however, as their habitat environment greatly affects where they are located, ticks are more frequently found in forested areas [3, 21, 22, 50, 62, 70]. Depending on the pathogen, the severity of tick-borne disease (TBD) can even be life-threatening. As human populations have grown, their interactions with the wild have increased. Additionally, human exposure to ticks carrying pathogens has greatly increased [55].

The transmission process of TBD is influenced by many factors, including pathogens, vectors, potential hosts, the environment, and human behavior. In addition, TBDs often benefit from human population mobility, animal migration, and global logistics. In particular, global logistics increase the possibility of tick-borne pathogens spreading across borders [65]. Worldwide, TBDs, such as tick-borne encephalitis, Crimean-Congolese hemorrhagic fever, and Rocky Mountain spotted fever, have posed new threats to public health worldwide, and the incidence of TBD is increasing at an alarming rate [34, 46]. The network of authorities in the United States reported nearly 650,000 cases of vector-borne diseases from 2004 to 2016. More than 75% of these cases were TBDs [48]. From 2016 to 2019, more than 200,000 cases of nine TBDs were reported to the Centers for Disease Control and Prevention (CDC). However, due to underreporting, the number of cases may be higher [48]. In addition, in mainland China, 33 new tick-borne diseases have been identified since the early 1980s, indicating a significant public health danger [64].

China is a large country with complicated topographical features, a varied climate, and several types of ticks. Therefore, TBDs are prevalent in most areas of China, seriously affecting human health [72]. Zhao *et al.* found that *D. nuttalli* is a tick species that carries many different tick-borne pathogens in China. According to a prediction model [71], the habitat suitable for 19 tick species was 14–476% larger than the geographical area where these species are currently found, indicating that there are still serious deficiencies in our knowledge of tick distribution. Due to large pasture areas and extensive animal husbandry, Inner Mongolia provides an excellent habitat for ticks, and the incidence of zoonosis is often very high [66]. In 2005, Jia *et al.* [17] reported human cases of *Rickettsia raoultii* in northeast China for the first time.

Ticks and TBDs have a substantial impact on the economy and human life in Inner Mongolia. Nevertheless, the community structure and diversity of microbial communities on ticks parasitizing sheep in various regions of Inner Mongolia have not been thoroughly studied thus far. The microbial communities of ticks are influenced by many factors, including geographical area, feeding status, blood meal source, and developmental stage [25, 49, 63].

Current techniques for 16S ribosomal RNA (rRNA) gene sequence analysis, based on typical clustering thresholds of operational taxonomic units (OTUs), are insufficient for accurate taxonomic annotation and for addressing phylogenetic relationships at the species level when only a few hypervariable regions are amplified. Therefore, amplified 16S rRNA gene sequence data of the V3–V4 region can only be explored at the genus level [45]. PacBio full-length 16S rDNA third-generation sequencing technology, however, is more accurate and can be used for reliable species identification [56].

Therefore, we applied this technology to analyze the microbial diversity of ticks collected in four main pastoral areas of Inner Mongolia. The aim of our investigation was to assess the distribution characteristics of tick microbial communities in different geographical locations in Inner Mongolia, providing information for better environmental management.

## Materials and methods

### Tick collection and sample preparation

**Figure 1:**
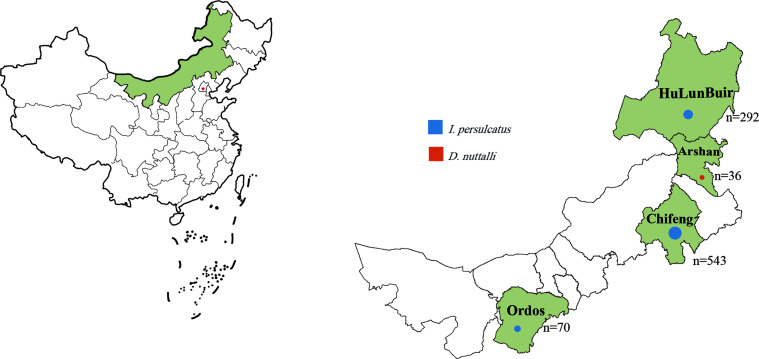
Map of tick collection sites in Inner Mongolia, China.

In mid-April 2019, adult ticks were collected with tweezers and stored in breathable bottles from bushes in the Arshan Forest area and from sheep pastures in Hulun Buir City (New Barag Right Banner), Chifeng City (Bayan Wendusumu area, Tianshan Town, Alukeerqin Banner), and Ordos City (Chengchuan Town, Otog Front Banner), Inner Mongolia (Fig. 1).

According to morphological identification based on the literature [15], the 905 ticks from Hulun Buir, Chifeng, and Ordos were all *D. nuttalli*. However, 36 ticks from bushes in the Arshan Forest area were identified as *I. persulcatus.* The morphological identification of the ticks was then double checked with molecular biology methods based on the sequences of the species-specific 16S rRNA gene [53, 59]. The 905 *D. nuttalli* and 36 *I. persulcatus* were stored at −80 °C until further processing.

### DNA extraction

The ticks were disinfected with 75% ethanol, dried on filter paper, washed three times with PBS, and finally dried on filter paper. A total of 941 ticks were divided into 206 sample pools. In each sample pool, 1–20 ticks were placed into a sterile grinding tube. One 5 mm magnetic bead and two 3 mm magnetic beads were added, and the grinding tube was placed into the adapter (cooled to −20 °C in a freezer for 30 min). The adapter was installed into the high-speed tissue homogenizer (Servicebio, Wuhan, China), and the parameters were set to 70 Hz and 180 s. Finally, according to the instructions for the tissue genomic DNA extraction kit (TIANGEN, Beijing, China), all extracted DNA was stored at −20 °C. Three DNA samples (Table 1) were randomly selected from each sample area, and 10 μL of each sample was prepared for microbial diversity sequencing.

**Table 1:** Information on tick samples used for bacterial microbiome analysis.

Sample name	Tick species	Number of ticks
Hulun Buir-1	*D. nuttalli*	8
Hulun Buir-2		1
Hulun Buir-3		1
Chifeng-1		1
Chifeng-2		1
Chifeng-3		8
Ordos-1		1
Ordos-2		1
Ordos-3		1
Arshan-1	*I. persulcatus*	6
Arshan-2		6
Arshan-3		6

### 16S rRNA amplicon sequencing

The V1–V9 regions of the bacterial 16S rRNA gene were amplified by PCR (95 °C for 2 min, followed by 27 cycles at 95 °C for 30 s, 55 °C for 30 s, and 72 °C for 60 s and a final extension at 72 °C for 5 min) using the primers 27F 5′–AGAGTTTGATCCTGGCTCAG–3′ and 1492R 5′–GGTTACCTTGTTACGACTT–3′ [39], where the barcode is an eight-base sequence unique to each sample. PCRs were performed in triplicate in a 20-μL mixture containing 4 μL of 5× FastPfu Buffer, 2 μL of 2.5 mM dNTPs, 0.8 μL of each primer (5 μM), 0.4 μL of FastPfu Polymerase, and 10 ng of template DNA. Amplicons were extracted from 2% agarose gels and purified using the AxyPrep DNA Gel Extraction Kit (Axygen Biosciences, Union City, CA, USA), according to the manufacturer’s instructions. SMRTbell libraries were prepared from the amplified DNA by blunt ligation according to the manufacturer’s instructions (Pacific Biosciences). Purified SMRTbell libraries from the pooled and barcoded samples were sequenced on dedicated PacBio Sequel cells using S/P1-C1.2 sequencing chemistry. Replicate 1 of the samples was sequenced using S/P2-C2/5.0 sequencing chemistry. Replicate 2 of the samples was sequenced with a prerelease version of the S/P3-C3/5.0 sequencing chemistry. All amplicon sequencing was performed by Shanghai Biozeron Biotechnology Co., Ltd. (Shanghai, China).

### Bioinformatic analysis of 16S rRNA amplicon sequence data

PacBio raw reads were processed using SMRT Link Analysis software version 6.0 to obtain demultiplexed circular consensus sequence (CCS) reads with the following settings: minimum number of passes = 3, minimum predicted accuracy = 0.99. Raw reads were processed through the SMRT Portal to filter sequences for length (<800 or >2500 bp) and quality. Sequences were further filtered by removing barcodes, primer sequences, chimeras and sequences that contained 10 consecutive identical bases. OTUs were clustered with a 97% similarity cutoff using UPARSE (RRID:SCR_005020), and chimeric sequences were identified and removed using UCHIME. The phylogenetic affiliation of each 16S rRNA gene sequence was analyzed with the RDP Classifier [7] (http://rdp.cme.msu.edu/) against the SILVA (RRID:SCR_006423) 16S rRNA database using a confidence threshold of 70% [2]. Subsequently, α and β diversity analyses were run. Rarefaction analysis based on Mothur (RRID:SCR_011947) [51] was used to reveal the Chao, ACE, and Shannon diversity indices. The β diversity analysis was performed using UniFrac (RRID:SCR_014616) [30] to compare the results of the principal coordinates analysis (PCoA) using the community ecology package R-forge (the vegan 2.0 package was used to generate a PCoA figure). One-way analysis of variance (ANOVA) tests were used to assess the statistically significant differences in diversity indices between samples. Differences were considered significant at *p* < 0.05. Venn diagrams were drawn using the online tool “Draw Venn Diagram” (http://bioinformatics.psb.ugent.be/webtools/Venn) to analyze overlapping and unique OTUs during the treatment processes.

For the identification of biomarkers for highly dimensional colonial bacteria, linear discriminant analysis effect size (LEfSe) was performed [52]. The Kruskal–Wallis sum rank test was applied to examine the changes and dissimilarities among classes followed by linear discriminant analysis (LDA) to determine the size effect of each distinctively abundant taxon [16].

### Phylogenetic analyses

Using Mega 7 software, sequences taken from the GenBank database were aligned with the ones obtained, and phylogenetic trees for the 16S rRNA gene were constructed using neighbor-joining (NJ) modeling to confirm the phylogenetic relationships of the pathogens discovered in this study. Bootstrap values for the individual branches of the resulting tree were determined by applying bootstrap analysis with 1,000 replicates. New sequences were deposited in GenBank with accession numbers OQ852069–OQ852073 and OP286852–OP286858.

## Results

### Identification of ticks

A total of 941 adult hard ticks were identified as *D. nuttalli* (*n* = 905) (accession number: OQ852069) and *I. persulcatus* (*n* = 36) (accession number: OQ852073) with molecular biology and morphological identifications. Two PCR products from each of the two tick species were randomly selected for sequencing comparison to confirm the morphological identifications, and both 16S rRNA gene sequences obtained were uploaded to the GenBank database.

### PacBio sequencing data

**Figure 2:**
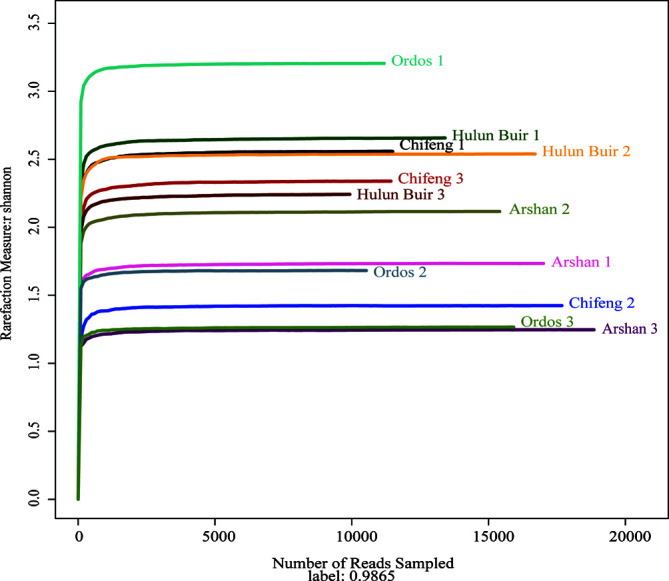
Shannon–Wiener curve. *X*-axis: amount of sequencing data; *Y*-axis: corresponding Shannon diversity index.

A total of 12 samples were sequenced (Table 1). After data screening and deletion, a total of 201,159 reads were generated and classified. The reads of each sample ranged from 11,550 to 21,996. The dilution curve of the Shannon index at the OTU level showed a suitable range for sequencing, and the observed Shannon index accumulation curve also reached a plateau (Fig. 2).

### Bacterial microbiome composition

**Figure 3:**
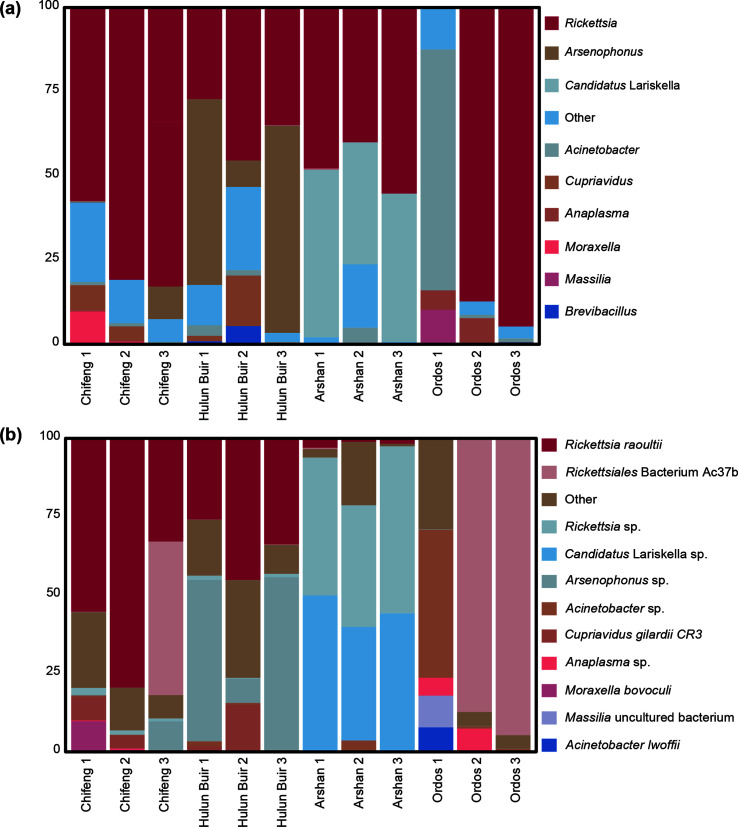
Microbe composition of different regions and species. (a) Top 10 microbial components at the genus level. (b) Top 12 microbial components at the species level.

A total of 326 OTUs were detected in 12 samples, and the bacterial microbial components were 11 phyla, 15 classes, 38 orders, 62 families, 104 genera, and 141 species. At the genus level (Fig. 3a), the abundance of *Rickettsia* was the highest at 54.60%, including *Rickettsiales* bacterium Ac37b (19.33%) and other *Rickettsia* (35.27%), followed by *Arsenophonus* (11.21%), *Candidatus* Lariskella (10.84%), *Acinetobacter* (7.17%), *Cupriavidus* (2.39%), and *Romboutsia* (1.00%). The most abundant *Rickettsia* were found in *D. nuttalli* in the Chifeng area, followed by *I. persulcatus* in the Arshan area, whereas *Candidatus* Lariskella was found primarily in *I. persulcatus* in Arshan. At the species level (Fig. 3b), *R. raoultii* had the highest abundance (23.23%), followed by *Rickettsiales* bacterium Ac37b (19.33%), *Rickettsia* sp*.* (11.94%), *Candidatus* Lariskella sp. (10.84%), and *Arsenophonus* sp. (10.32%). *Rickettsia raoultii* was found in samples of *I. persulcatus* and the majority of *D. nuttalli*, except in Ordos. Most *Rickettsiales* bacterium Ac37b was found in *D. nuttalli* of Ordos and Chifeng, and was most prevalent in Ordos.

### Differences in ticks in different areas

**Figure 4:**
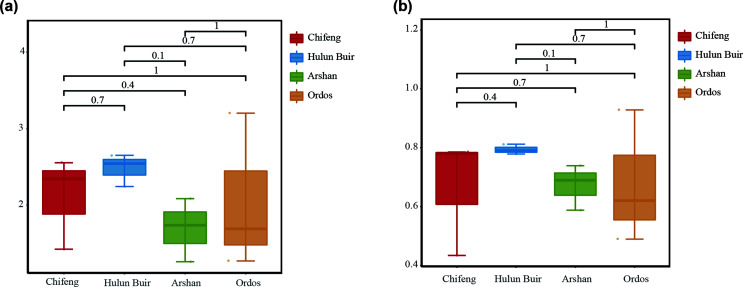
Alpha diversity measures for *Dermacentor nuttalli* and *Ixodes persulcatus* in four areas. (a) Shannon’s index. (b) Simpson’s index.

**Figure 5:**
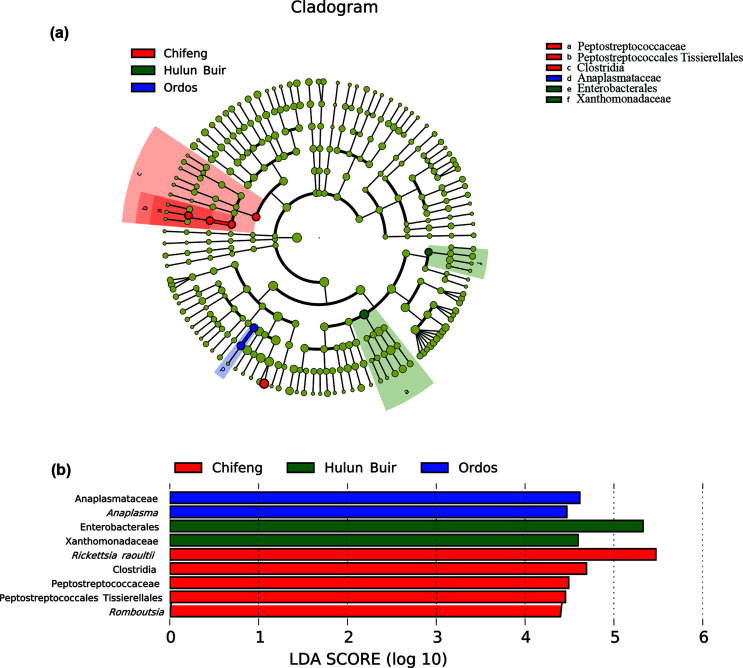
(a) Clustering tree analysis by linear discriminant analysis effect size (LEfSe). (b) Histogram of LDA analysis.

**Figure 6:**
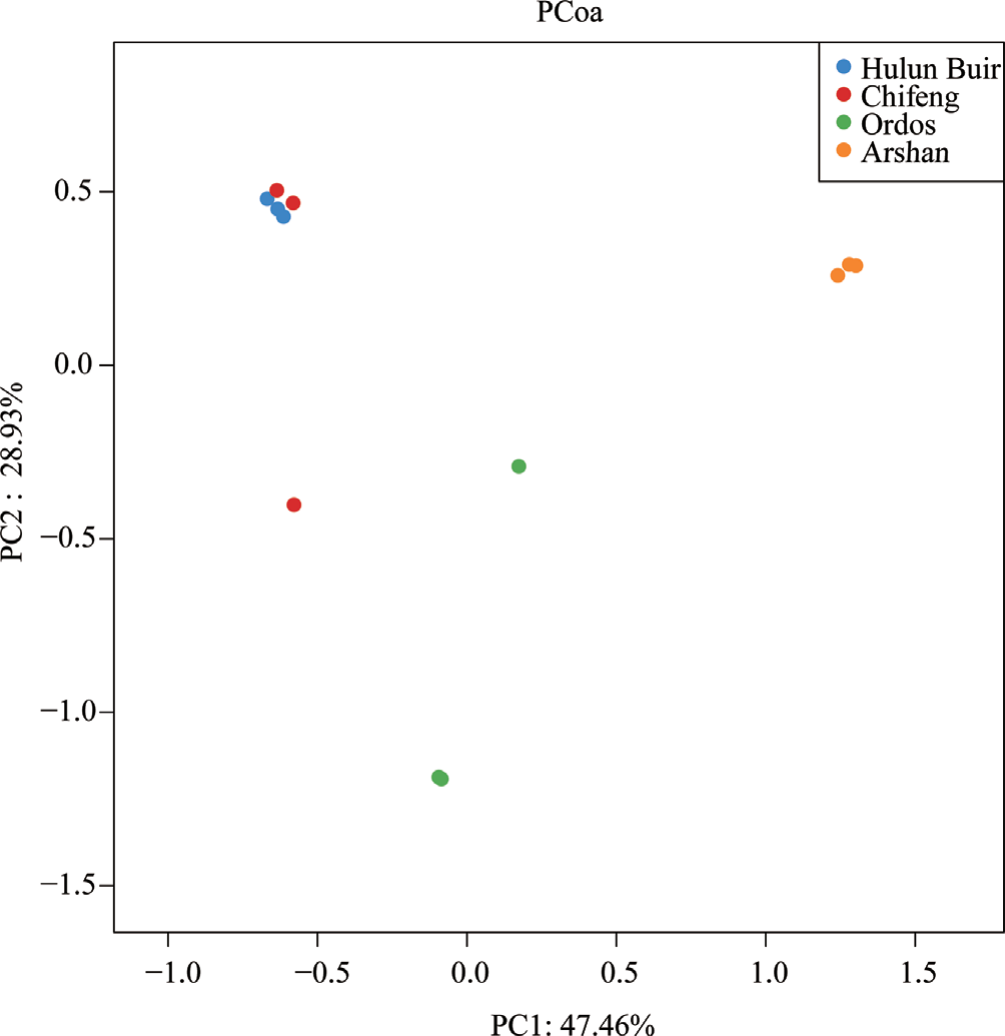
PCoA of β-diversity measures for twelve groups. Weighted UniFrac PCoA graph showing PC1, which accounts for 47.46% of variation, and PC2, which accounts for 28.93% of variation. Different colored dots represent different regions and species.

There were 180, 182 and 135 OTUs in the samples of *D. nuttalli* from Hulun Buir, Chifeng and Ordos, respectively. According to the α diversity, the Hulun Buir region showed greater microbial diversity than the other two regions (Fig. 4). LEfSe showed that *R. raoultii*, Peptostreptococcaceae, and *Clostridia* played an important role in the Chifeng area. *Anaplasma* was characteristic of the Ordos formation, while Enterobacterales and Xanthomonadaceae were found at Hulun Buir (Fig. 5). To further distinguish the composition of the microbial community, weighted UniFrac analyses revealed differences regarding the region, as measured by an analysis of similarity (ANOSIM, *R* = 0.7994, *p* = 0.011) and visualized by PCoA (Fig. 6). PCoA explained 47.46% (Axis 1) and 28.93% (Axis 2) of the variation, with samples from different regions clustering separately (Fig. 6).

### Differences between *Dermacentor nuttalli* and *Ixodes persulcatus*

**Figure 7:**
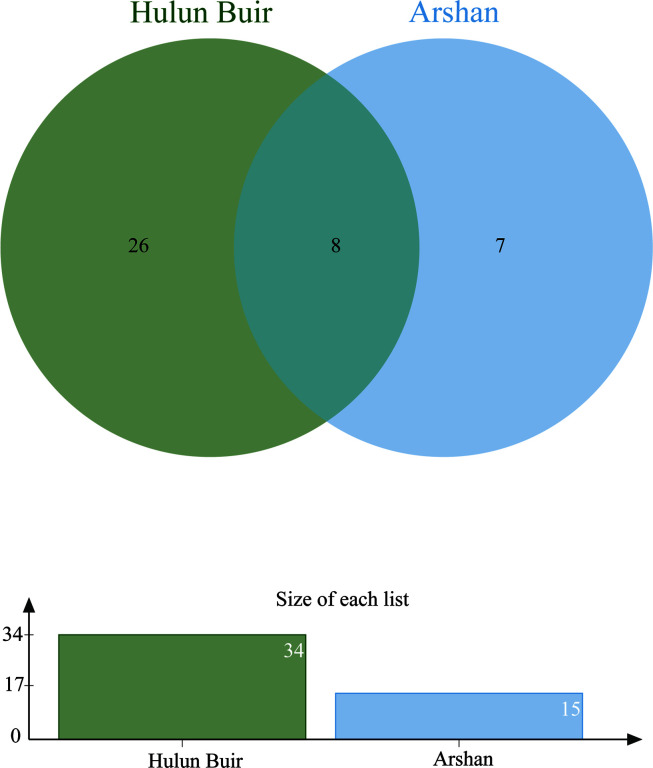
Venn diagram for cluster analysis of OTUs between *Dermacentor nuttalli* and *Ixodes persulcatus* (genus level).

North of the Arshan region is adjacent to the New Barag Left Banner and to the Ewenki Autonomous Banner of Hulun Buir City, close to the Hulun Buir tick collection point. All *I. persulcatus* came from the Arshan region. In the Hulun Buir area, only *D. nuttalli* was found. A total of 123 microbial OTUs were found in ticks, and α diversity indicated that specimens of *D. nuttalli* contained greater microbial diversity than specimens of *I. persulcatus* (Fig. 4). Analysis of OTU clusters at the genus level of *D. nuttalli* and *I. persulcatus* samples found that their most common bacteria were *Acinetobacter*, *Cutibacterium*, *Pseudomonas*, *Ralstonia, Rhodanobacter*, *Rickettsia* and *Vibrionimonas* (Fig. 7).

### Analysis of major bacteria and pathogenic bacteria

**Figure 8:**
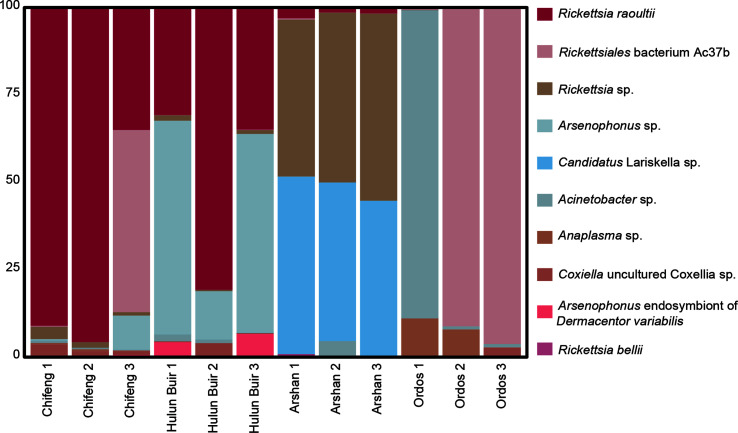
The composition of bacteria and pathogenic bacteria with a high abundance.

We selected bacteria with high abundance and obvious harm to humans at the species level (*R. raoultii*, *Anaplasma* and *Coxiella*) for further analysis (Fig. 8).

### Phylogenetic analysis of *Rickettsiales* bacterium Ac37b

**Figure 9:**
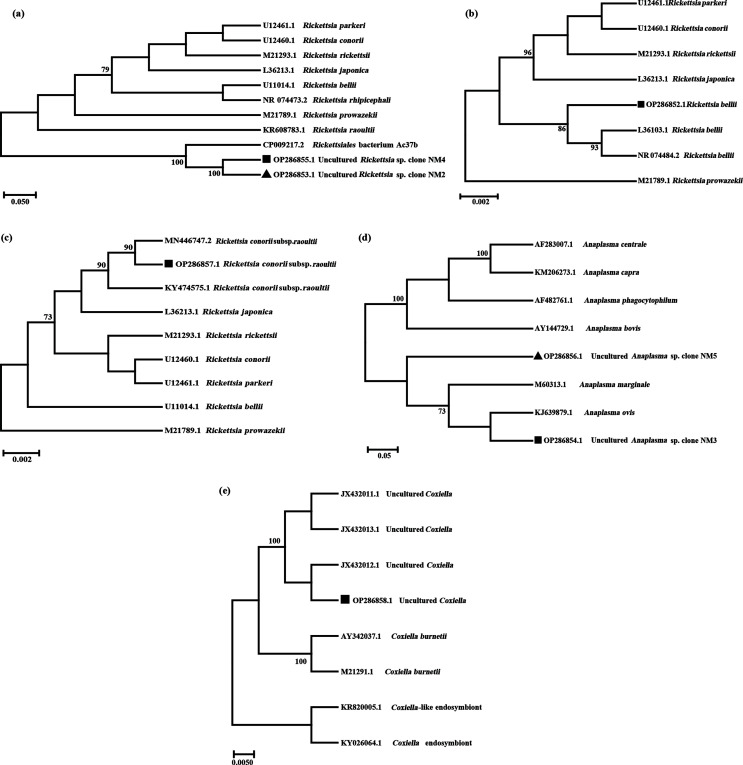
Phylogenetic tree of *Rickettsiales* bacterium Ac37b, *Rickettsia bellii*, *Rickettsia raoultii*, *Anaplasma* and *Coxiella* in ticks based on neighbor-joining (NJ) modeling; only values higher than 60 were added to the tree branches. (a) Phylogenetic tree of *Rickettsiales* bacterium Ac37b identified in Inner Mongolia; the 16S rRNA gene sequences obtained in this study are marked with black squares (1320 bp) and triangles (1430 bp). (b) Phylogenetic tree of *Rickettsia bellii* identified in Inner Mongolia; the 16S rRNA gene sequences obtained in this study are marked with black squares (1109 bp). (c) Phylogenetic tree of *Rickettsia raoultii* identified in Inner Mongolia; the 16S rRNA gene sequences obtained in this study are marked with black squares (855 bp). (d) Phylogenetic tree of *Anaplasma* identified in Inner Mongolia; the 16S rRNA gene sequences obtained in this study are marked with black squares (1455 bp) and triangles (547 bp). (e) Phylogenetic tree of *Coxiella* identified in Inner Mongolia; the 16S rRNA gene sequences obtained in this study are marked with black squares (1463 bp).

The *Rickettsiales* bacterium Ac37b (OP286853 and OP286855) sequences were randomly selected to build a phylogenetic tree, named after regions and displayed on the phylogenetic tree with squares and triangles (Fig. 9a). The phylogenetic tree of the 16S rRNA gene showed that *Rickettsiales* bacterium Ac37b found in Inner Mongolia is in the same branch as the *Rickettsiales* bacterium Ac37b (CP009217) found in *Amblyomma cajennense* from Brazil, and their homology is 100% according to sequence alignment analysis using MEGA 7.0 software.

### Phylogenetic analysis of *Rickettsia bellii*

*Rickettsia bellii* (OP286852) was found only in *I. persulcatus*, and the sequence of *R. bellii* was randomly selected to construct the evolutionary tree. The sequence of *R. bellii* obtained in this study is displayed on the phylogenetic tree with squares (Fig. 9b). The phylogenetic tree of the 16S rRNA gene shows that *R. bellii* found in Inner Mongolia is on the same branch as *R. bellii* strain RML369-C (NR074484). The sequences were compared and analyzed with MEGA 7.0 software and showed that the 16S rRNA gene sequence of *R. bellii* from Inner Mongolia showed 94.23% homology with the *R. bellii* strain RML369-C (NR074484) isolated in Marseille, France.

### Phylogenetic analysis of *Rickettsia raoultii*

The *R. raoultii* (OP286857) sequences were randomly selected to build a phylogenetic tree, named after regions and displayed on the phylogenetic tree with squares (Fig. 9c). The phylogenetic tree of the 16S rRNA gene shows that *R. raoultii* found in Inner Mongolia is on the same branch as *R. raoultii* strain IM16 (KY474575) and *R. raoultii* isolate Binxian-91 (MN446747). The sequences were compared and analyzed with MEGA 7.0 software and show that the 16S rRNA gene sequence of *R. raoultii* in Inner Mongolia is 100% homologous with the *R. raoultii* IM16 strain (KY474575) in China and *R. raoultii* (MN446747) found in *Haemaphysalis longicornis* in Bin County, Shanxi Province.

### Phylogenetic analysis of *Anaplasma*

The *Anaplasma* (OP286854 and OP286856) sequences were randomly selected to build a phylogenetic tree, named after regions and displayed on the phylogenetic tree with squares and triangles (Fig. 9d). The phylogenetic tree of the 16S rRNA gene shows that *Anaplasma* found in Inner Mongolia is on the same branch as *Anaplasma ovis* strain Ao89 (KJ639879). The sequences were compared and analyzed using MEGA 7.0 software. They show that the 16S rRNA gene sequence of *Anaplasma* in Inner Mongolia has 99.93% homology with *A. ovis* strain Ao89 (KJ639879) found in the Qilian Mountains, Lanzhou.

### Phylogenetic analysis of *Coxiella*

The *Coxiella* (OP286858) sequences were randomly selected to build a phylogenetic tree, named after regions and displayed on the phylogenetic tree with squares (Fig. 9e). The phylogenetic tree of the 16S rRNA gene shows that *Coxiella* found in Inner Mongolia is on the same branch as uncultured *Coxiella* sp. clones DX-56 (JX432012) and DX-68 (JX432013). The sequences were compared and analyzed using MEGA 7.0 software. The results show that the homologies between the 16S rRNA gene sequence of *Coxiella* in Inner Mongolia and the sequences of *Coxiella* DX-56 (JX432012) and *Coxiella* DX-68 (JX432013) are 99.93% and 99.66%, respectively.

## Discussion

*Dermacentor nuttalli* is widely distributed in northern China. This tick is parasitic on livestock and causes severe disease in humans [24, 60]. The species carries different pathogens, such as *Babesia*, *A. ovis*, *Rickettsia*, and *Coxiella* [41, 57, 67]. *Ixodes persulcatus* is the dominant tick species in northeast China. Because of the large east–west span of Inner Mongolia, *I. persulcatus* populations exist at the border with northeast China [33]. The species is usually associated with eight species of viruses, including Alongshan virus and tick-borne encephalitis virus, which pose a serious threat to human health and safety [61].

*Dermacentor nuttalli* is the dominant tick species in Inner Mongolia, with a major influence on the economy and health of the local human population. Jiao *et al.* [18] carried out a simple microbial diversity analysis of ticks on cattle in the Hulun Buir area of Inner Mongolia. Beyond this, the microbial community composition of ticks in other areas of Inner Mongolia was not further investigated. The bacterial diversity of different tick species must be further analyzed to better understand the relationships between ticks and microorganisms. Samples collected from multiple regions are more likely to harbor new pathogens in their microbial diversity.

In our investigation, we applied PacBio full-length 16S rRNA third-generation sequencing to the V1–V9 regions of the 16S rRNA. In their study on oral microorganisms, Zhang *et al.* [69] found that OTU sequences generated by PacBio were much larger than those generated on the MiSeq platform. One of the advantages of third-generation sequencing is that it enables direct reading of DNA molecules without the need for PCR amplification or library construction. By avoiding these steps, third-generation sequencing can provide more realistic, comprehensive, and high-quality genome sequences [58]. Therefore, we adopted PacBio full-length 16S rRNA third-generation sequencing for the microbial diversity analysis of ticks in Inner Mongolia. According to our analysis, the microbial diversity in *D. nuttalli* and *I. persulcatus* samples from different regions of Inner Mongolia differed. The highest microbial diversity was discovered in *D. nuttalli* samples from Hulun Buir, where the main constituent microorganisms were Rickettsia (35.92%) and Arsenophonus (41.51%). *Rickettsia* is an arthropod-associated obligate intracellular gram-negative bacterium that can cause mild to severe disease in humans [10, 47]. *Arsenophonus* is an intracellular symbiotic bacterium of insects with a wide host range and rich biodiversity [35]. The “son killer” of the parasitic wasp *Nasonia vitripennis* is due to the male-killing phenotype [40]. An effect on ticks has not yet been reported, and its other biological functions have not yet been identified [29]. Studies have shown that the microbiological makeup of ticks can be influenced by a variety of factors, including tick species, life stage, sex, host blood feeding, and geographic location [23]. It is possible that the geographic location and the small number of *I. persulcatus* samples used in this study contributed to the increased microbial diversity discovered in *D. nuttalli* compared with *I. persulcatus*. In China, 28 species of human pathogens have been detected in *I. persulcatus* [61]. Using PacBio full-length 16S rRNA third-generation sequencing, only the microbial composition of bacteria in ticks was investigated in this study. Although third-generation sequencing technology has excellent coverage and read length, due to its relatively high single-molecule error rate, deep sequencing is required to obtain high-quality data results. Second-generation sequencing technologies can provide an additional level of validation, as they have higher precision and accuracy than third-generation sequencing and can detect low-frequency variants. PCR-based methods can also be used to validate the results and provide an additional level of confidence. Combining the results of multiple methods can help to improve the accuracy and reliability of pathogen identification.

*Rickettsia raoultii* is a pathogen of the spotted fever group, which is transmitted vertically in arthropods as a symbiotic bacterium and in vertebrates as a pathogenic bacterium of human diseases [11]. *Anaplasma* is a gram-negative intracellular obligate parasite, and its pathogenicity poses an important threat to several animal species and to public health [4]. Currently, there are six species of *Anaplasma* recognized worldwide, *i.e.*, *A. phagocytophilum*, *A. ovis*, *A. capra*, *A. bovis*, *A. marginale,* and *A. platys* [20]. In addition to *A. phagocytophilum*, *A. bovis* and *A. capra* have been reported to infect humans [6, 28]. A novel typhus group *Rickettsia* species, *Rickettsiales* bacterium Ac37b, was found in the Ordos and Chifeng region [54]. The *Rickettsia* typhus group is composed of *R. prowazekii* and *R. typhi*. In Australia, three types of typhus, epidemic typhus, murine typhus, and tsutsugamushi disease (scrub typhus), have been found successively. These are closely related to native wild animals and ticks in Australia [13]. In China, scrub typhus has also been an important cause of human morbidity and mortality in the past decade. The disease was initially identified only in southern China, but now cases of typhus have been reported in northern China, with a wide geographical distribution [31]. Notably, *Candidatus* Lariskella was found in *I. persulcatus* in Arshan. *Candidatus* Lariskella was initially proposed in 2012 [36]. “*Candidatus* Lariskella arthropodarum” was found in samples taken from individuals in Russia who had acute febrile illnesses as well as in locally obtained *I. persulcatus* [38], showing its potential pathogenicity.

Through the construction of phylogenetic trees using 16S rRNA genes of the major bacteria *R. bellii* and main pathogenic bacteria *Rickettsiales* bacterium Ac37b, *Anaplasma*, *Coxiella*, and *R. raoultii* from this study, it was found that *R. bellii* showed a 94.23% homology with the *R. bellii* strain RML369-C (NR074484) isolated from France, *Rickettsiales* bacterium Ac37b had 100% homology with *Rickettsiales* bacterium Ac37b (CP009217) isolated from Brazil, *Anaplasma* had high homology with *A. ovis* strain Ao89 (KJ639879) in the Qilian Mountains (99.93%), *Coxiella* was highly homologous with *Coxiella* DX-56 (JX432012) (99.93%), and *R. raoultii* had 100% homology with *R. raoultii* strain IM16 (KY474575). The Qilian Mountains and Hebei are both in northern China, and Inner Mongolia is close to them. Therefore, the genetic evolution of tick-borne pathogens may be similar. Among them, both symbiotic bacteria and pathogens of the *Coxiella* genus can cause disease and lead to the death of humans and animals, while *Coxiella burnetii* is the pathogen causing Q fever [9]. However, at present, it is not clear whether ticks are of great significance in the natural transmission of *C. burnetii* [5]. *Rickettsia bellii* is the only known species in a third group that differentiated before the spotted fever group and the typhus group separated. *Rickettsia bellii* is the most prevalent *Rickettsia* species in American ticks, and it has been detected in a wide range of tick species, exhibiting the broadest arthropod host range among all known *Rickettsia* [26, 27, 44]. It causes slight inflammatory reactions in mammals [42]. However, the pathogenic potential for humans is still unknown and should be closely monitored [1].

It is necessary to further explore the relationship between *R. bellii* and other *Rickettsia*. In the Ordos area, pathogenic bacteria that are more threatening to humans were found, and a new classification of *Rickettsia* emerged. Pathogen prevention and control in this area needs to focus on monitoring and strengthening the popular knowledge of methods for personal protection. The newly discovered pathogens in Inner Mongolia need to be isolated and sequenced in future studies, and the pathogenicity of the organisms should be tested through subsequent animal experiments.

As essential vectors for carrying and spreading infections, ticks have a significant impact on the health of humans and other terrestrial vertebrates. Microbiota vaccines are used against endosymbiotic and/or nonendosymbiotic bacteria found in ticks and can help to stop the spread of ticks and/or tick-borne illnesses [63]. The findings of this study offer fundamental information for the development of a tick microbiota vaccine to stop the spread of infections carried by ticks in the Inner Mongolia Autonomous Region.

In this study, we analyzed the microbial diversity of two ticks in four regions of Inner Mongolia. A novel *Rickettsia* species, *Rickettsiales* bacterium Ac37b, was found in Inner Mongolia for the first time, and *R. bellii* was found in *I. persulcatus*. Ticks carried more potential pathogens in the Ordos area, and there were coinfections of *Rickettsia* and *Anaplasma*, which may be related to the geographical environment. Although the number of ticks collected was limited, we unveiled the microbial diversity in ticks in Inner Mongolia using third-generation sequencing. The findings of our study indicate that certain pathogens identified in ticks have the potential to cause severe human diseases. These results have major implications for researchers investigating tick-borne illnesses and can aid in the development of preventive and therapeutic strategies.
